# Super-strong and Intrinsically Fluorescent Silkworm Silk from Carbon Nanodots Feeding

**DOI:** 10.1007/s40820-019-0303-z

**Published:** 2019-09-11

**Authors:** Suna Fan, Xiaoting Zheng, Qi Zhan, Huihui Zhang, Huili Shao, Jiexin Wang, Chengbo Cao, Meifang Zhu, Dan Wang, Yaopeng Zhang

**Affiliations:** 1 State Key Laboratory for Modification of Chemical Fibers and Polymer Materials, Shanghai Belt and Road Joint Laboratory of Advanced Fiber and Low-Dimension Materials, College of Materials Science and Engineering, Donghua University, Shanghai, 201620 People’s Republic of China; 2 State Key Laboratory of Organic-Inorganic Composites, Beijing University of Chemical Technology, Beijing, 100029 People’s Republic of China; 3 School of Chemistry and Chemical Engineering, Shandong University, Jinan, 250100 People’s Republic of China; 4 School of Chemistry and Chemical Engineering, Yantai University, Yantai, 264005 People’s Republic of China

**Keywords:** Silkworm silk, Carbon nanodot, Mechanical property, Fluorescent silk, Feeding method

## Abstract

Fluorescent silk is fundamentally important for the development of future tissue engineering scaffolds. Despite great progress in the preparation of a variety of colored silks, fluorescent silk with enhanced mechanical properties has yet to be explored. In this study, we report on the fabrication of intrinsically super-strong fluorescent silk by feeding *Bombyx mori* silkworm carbon nanodots (CNDs). The CNDs were incorporated into silk fibroin, hindering the conformation transformation, confining crystallization, and inducing orientation of mesophase. The resultant silk exhibited super-strong mechanical properties with breaking strength of 521.9 ± 82.7 MPa and breaking elongation of 19.2 ± 4.3%, improvements of 55.1% and 53.6%, respectively, in comparison with regular silk. The CNDs-reinforced silk displayed intrinsic blue fluorescence when exposed to 405 nm laser and exhibited no cytotoxic effect on cells, suggesting that multi-functional silks would be potentially useful in bioimaging and other applications.

**Electronic supplementary material:**

The online version of this article (10.1007/s40820-019-0303-z) contains supplementary material, which is available to authorized users.

## Highlights

- Intrinsically, super-strong fluorescent silk was fabricated via feeding *Bombyx mori* silkworms with carbon nanodots.
- The multi-functional silks showed no cytotoxicity to Schwann cells and exhibited great potential in bioimaging.
- The reinforcing mechanism of multi-functional silks was proposed.

**Electronic supplementary material:**

The online version of this article (10.1007/s40820-019-0303-z) contains supplementary material, which is available to authorized users.

## Graphical abstract

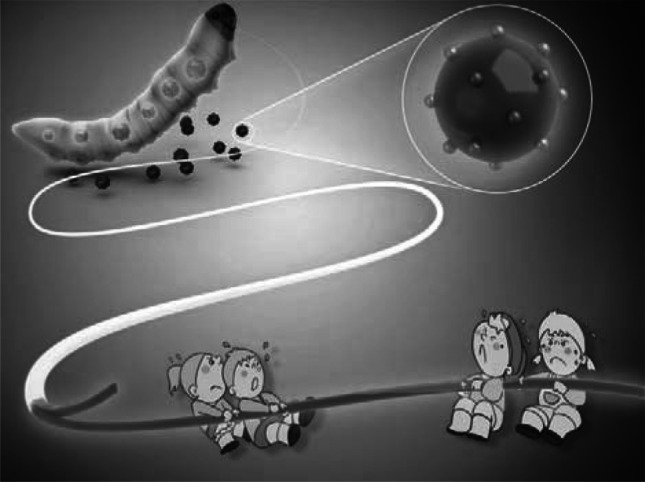

## Introduction

Fluorescent silk, one of the novel natural functional biomaterials, is gaining enormous attention due to its great potential for biomedical and intelligent textile-related applications [1–5]. It has been reported that fluorescence can be imparted through the incorporation of various organic dyes and inorganic nanoparticles into silk through post-dyeing of naturally produced silk [6, 7]. This method inevitably requires harsh post-processing conditions and complex procedures, which may devastate the original properties of the silk. Alternatively, modification of silkworm genes to obtain intrinsically fluorescent silk has been reported [8–11]. This technique has great potential for the production of bio-functional silk in the future, especially as the mass production of spider silk can be achieved through targeted gene replacement in *Bombyx mori* [12]. Furthermore, a recent study has reported the direct production of intrinsically fluorescent silk from silkworms fed on a diet containing dye [13]. Compared with other methods, feeding method is considerably more convenient and environmentally friendly since it eliminates all external dyeing processes and reduces the use of the resources associated with it.

Besides its fluorescent capability, the properties required for fluorescent silk for use in biomedical scaffolds and diagnostic and therapeutic devices are excellent mechanical properties and lack of cytotoxicity. To date, to the best of our knowledge, the majority of fluorescent silk only restores the original mechanical properties of silk, which is still much weaker than that of spider dragline silk or other reinforced silk [14, 15]. Super-strong silk with intrinsically fluorescence has not yet been reported.

In the present study, we have reported the fabrication of reinforced and fluorescent multi-functional silk produced directly by silkworms, that is, reinforced intrinsically fluorescent silk, and studied its physical properties. Based on the reinforcing mechanism, we hypothesize that the fluorescent material added to the silkworms’ diet should present a number of functional groups that would be able to interact with silk fibroin, but otherwise have little effect on the original structure of silk fibroin. As potential candidates, it was found that carbon nanodots (CNDs) have the desired structure [16, 17]. Furthermore, as a nanodye, CNDs disperse homogeneously in the diet, favoring in vivo uptake to form high-quality silk [18]. In addition, CNDs are a new class of fluorescent materials that has attracted much attention for applications in biomedical field owing to their adjustable parameters, good fluorescence stability, and excellent biocompatibility [19–21].

## Experimental

### Carbon Nanodots

Carbon nanodots (CNDs) were provided by Professor D. Wang, Beijing University of Chemical Technology, and prepared using a modified hydrothermal method with citric acid and ethylenediamine as precursors [22]. The CNDs exhibited excellent dispersibility in aqueous solution and had diameters ranging from 1 to 5 nm (Fig. S2).

### Preparation of CNDs-Modified Artificial Diet

The *Bombyx mori* eggs were provided by the Sericultural Research Institute of Guangxi, China. The artificial silkworm diet was purchased from Shandong Sericultural Research Institute, China. The preparation of artificial diet is the same as our previous work [23]. Dry diet power was mixed uniformly with CNDs in aqueous suspension, followed by microwaving for 5 min, and then pressed into wafer. The mass fraction ratio of CNDs/dry diet powder was 0.00, 0.75, 1.00, and 1.25%.

### Raising of Silkworm

The *Bombyx mori* silkworms were raised in a climatic chamber. The humidity and temperature in different growth periods were controlled accurately as previously reported by Cai et al. [23]. All silkworm larvae were fed with normal artificial diet prior to the second day of the fifth instar. A total of 100 silkworms were then allocated equally into four groups. Three groups were fed a CNDs-modified diet from the second day of the fifth instar to the start of spinning since the growth of silk gland and biosynthesis and secretion of silk fibroin occurred mostly during this period [24]. The other group was fed a normal artificial diet over the whole study. Finally, the survival rate of each group was up to 95%. The silkworms and resultant silks obtained were termed CNDs-0.75%, CNDs-1%, CNDs-1.25%, and control, respectively.

The normal artificial diet consisted of mulberry leaf powder (38.4%), defatted soybean powder (36.9%), corn powder (9.0%), agar powder (5.0%), green branches and petioles powder (5.0%), other trace substances, including vitamin C, vitamin B complex, choline chloride, and citric acid.

### Cocoon Degumming

Prior to degumming, the obtained cocoons were dried at 100 °C for 60 min and at 80 °C for 180 min in a vacuum drying oven. The cocoons were then degummed three times in boiling 0.5 wt% Na_2_CO_3_ aqueous solution for 30 min and rinsed with distilled water. Finally, the degummed silks were dried at room temperature.

### Cell Culture and Characterization

Silk scaffolds were constructed by winding silk fibers around a hollow plastic frame (Fig. 4a), followed by degumming. Silk scaffolds were placed into 24-well plate and sterilized using 75% (v/v) ethanol aqueous solution and UV light, followed by washing with phosphate-buffered saline (PBS) three times. Schwann cells (SCs) were obtained from the Institute of Biochemistry and Cell Biology of the Chinese Academy of Sciences (Shanghai, China). They were seeded on scaffolds at a density of 1 × 10^4^ cells per well and cultured in Dulbecco’s modified Eagle medium (DMEM) supplemented with 10% (v/v) fetal calf serum and 10% (v/v) double antibody in a cell incubator at 37 °C in an atmosphere containing 5% CO_2_. Culture medium was exchanged every 2 days. Cell viability was evaluated using 3-[4,5-dimethyl-2-thiazolyl]-2,5-diphenyl-2H-tetrazolium bromide (MTT) method after seeding for 2, 4, and 6 days. After 4 days, scanning electron microscopy (SEM, S-4800, Japan) and laser scanning confocal microscopy (LSCM, TCS SP5, Germany) were used to study the morphology of SCs. For observation by SEM, SCs seeded on scaffolds (SCs/scaffolds) were washed with PBS three times and then fixed in 2.5% paraformaldehyde at 4 °C for 2 h. After removing paraformaldehyde, SCs/scaffolds were washed in PBS three times again, followed by dehydration through a gradient of ethanol aqueous solutions (30, 50, 70, 75, 80, 90, and 100 vol%). Finally, the resulting SCs/scaffolds were freeze-dried in tert-butanol solution. SEM images were obtained at 10 kV. For observation by LSCM, SCs/scaffolds were fixed in 4.0% paraformaldehyde for 10 min, followed by washing in PBS three times and permeabilizing using 0.1% Triton X-100 solution for 5 min. The samples were blocked in 1.0% BSA solution for 20 min. Finally, SCs/scaffolds were incubated with phalloidin solution for 30 min. The laser wavelength was 405 nm.

### Structural Characterization

Silk surface morphology was imaged using a Hitachi S-3000 N scanning electron microscope (SEM). The CNDs were observed using a JEM-2100 transmission electron microscope (TEM) operated at 200 kV. Photoluminescence (PL) spectra following excitation using a 370 nm laser were collected on JASCO FP-6600 PL instrument. The CLSM (confocal laser scanning microscopy) images of the degummed silk were obtained after excitation with a 405 nm laser and acquired on TCS SP5 LSCM. 3D CLSM images were reconstructed using Imaris software.

Fourier transform infrared (FTIR) spectra were recorded on a Nicolet 6700 Fourier transform spectrometer attenuated total reflectance (ATR) accessory. Quantitative analysis of secondary structure was conducted by spectra deconvolution of amide I band [25, 26].

Synchrotron radiation wide-angle X-ray diffraction (SR-WAXD) was performed on BL15U1 beamline at Shanghai Synchrotron Radiation Facility. The wavelength (λ) and the spot size of the X-ray were 0.07746 nm and 3 × 2 μm^2^, respectively. FIT2D (V12.077) software and Peakfit (V4.12) software were utilized to process data obtained from these analyses. The process method was described in detail in our previous work [27].

The diameter of degummed silk was measured using an Olympus BX-51 optical microscope. However, the cross section of silk was irregular, and a more accurate diameter was confirmed through a comparison of the method described above and typical weight/length method, as detailed in Ref. [23]. For each sample, more than 15 single silk fibers were measured. Subsequently, the mechanical properties of silks were measured on Instron 5565 at 25 °C and (45 ± 5)% relative humidity. The extension rate and the gauge length were 2 mm min^−1^ and 10 mm, respectively.

## Results and Discussion

### Growth of Silkworms and Physical Properties of Cocoons

**Fig. 1:**
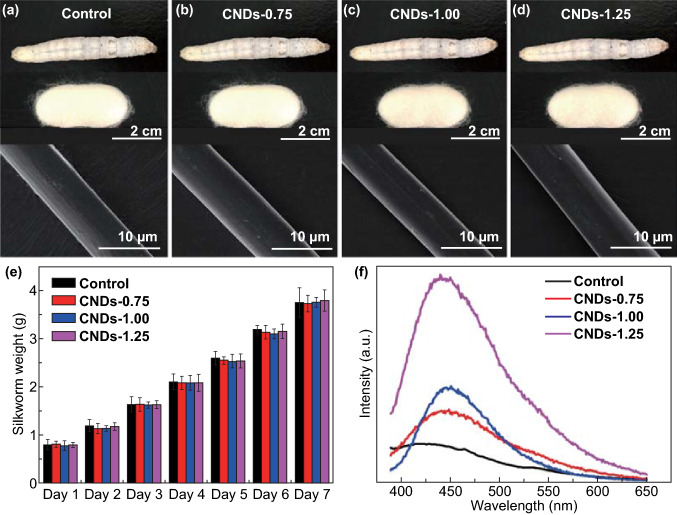
Preparation of multi-functional silk from silkworms fed CNDs. **a**–**d** Photographs of mature larvae fed different diets, corresponding cocoons and degummed silks. **e** Weight of silkworm larvae of the fifth instar from first to seventh day. **f** PL spectra of degummed silks measured using an excitation wavelength of 370 nm

The *Bombyx mori* larval silkworms were raised in the climatic chamber by feeding with CNDs-modified diet from the second day of the fifth instar to the start of spinning, in order to produce multi-functional silks. The diets containing different content CNDs were prepared as previously described [23]. The cocoons and silk fibers obtained by feeding diets with CNDs concentration of 0, 0.75, 1.00, and 1.25 wt% were named by control, CNDs-0.75, CNDs-1.00, and CNDs-1.25, respectively. All silk larvae had a similar weight over the duration of fifth instar (Fig. 1e) with no differences between the control mature larva and those fed with CNDs-modified diet. This indicated that modified diets prepared in this study were safe for silkworm. In addition, the cocoons obtained in this way exhibited similar colors and sizes (Fig. 1a–d). After drying, the cocoons were boiled in 0.5 wt% Na_2_CO_3_ aqueous solution to remove sericin so as to obtain degummed silks, used for subsequent characterizations. Figure 1a–d demonstrates that degummed silk exhibited similar smooth morphology with a diameter of 7 µm. No any CNDs were observed, illustrating that direct feeding with modified diet had no apparent effects on silk morphology. This may be attributed to the small number and excellent water dispersibility of CNDs (Fig. S2), a characteristic difference from graphene, TiO_2_, and other inorganic nanoparticles.

### Fluorescent Properties of CNDs-Modified Silks

**Fig. 2:**
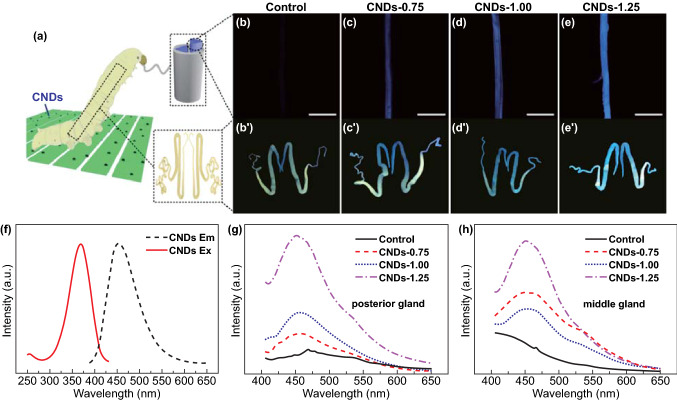
Influence of CNDs additives on the intrinsic fluorescent intensity of silk. **a** Schematic of silkworm fed a CNDs-modified diet and the corresponding silk gland and silk fiber. **b**–**e** Confocal laser scanning microscopy images of degummed silks (measured with an excitation wavelength of 405 nm). Scale bar represents 50 μm. **b**′–**e**′ Ultraviolet (UV) pictures of silk glands of mature larvae measured under a UV lamp. **b**, **b**′ Control, **c**, **c**′ CNDs-0.75%, **d**, **d**′ CNDs-1%, **e**, **e**′ CNDs-1.25%. **f**–**h** PL spectra of (**f**) CNDs, (**g**) posterior and (**h**) middle silk glands with an excitation wavelength of 370 nm

An important observation was that just a small quantity of CNDs could endow the silk with unique properties. As shown in the PL spectra (Fig. 1f), the modified silks exhibited a strong emission peak at 450 nm, the intensity of which increased as the concentration of CNDs increased. The control silks demonstrated only a weak but broad emission peak centered at 420 nm. The CNDs in the present study exhibited a strong emission peak centered at 450 nm (Fig. 2f); hence, we concluded that the fluorescent property originated from the CNDs, and silkworms could take in a certain quantity of CNDs.

As a result, all degummed silks, except the controls, exhibited an intrinsic homogeneous blue fluorescence at an excitation wavelength of 405 nm (Figs. 2b–e and S3). The CNDs-1.25 silk exhibited the brightest blue fluorescence, consistent with the PL spectra (Fig. 1f). It is worth noting that samples with blue fluorescence were degummed silk rather than cocoon, indicating that the fluorescence originated from silk fibroin brin. This was different from the naturally colored silk produced by wild silkworms, the color of which emanated from sericin rather than fibroin, and was lost after degumming [13].

In order to further ascertain the origin of blue fluorescence of the silk, the silk glands of mature larvae fed with different diets were compared under UV lamp. The whole silk glands of control silkworm exhibited a light yellowish green (Fig. 2b′) fluorescence, while the middle and anterior silk gland of CNDs-0.75 silkworm fluoresced light blue although the color of the fluorescence from the posterior silk gland (Fig. 2c′) was similar to that of control silkworm. As the content of CNDs reached up to 1 and 1.25 wt%, the whole silk glands fluoresced deep blue (Fig. 2d′–e′) and further confirmed by the PL spectra of middle and posterior silk glands (Fig. 2g–h). In addition, the color of the fluorescence of glands became bluer with the concentration of silk fibroin from posterior silk gland to anterior division of middle silk gland. Asukura [28] pointed out that silk fibroin was polymerized in posterior silk gland. These observations illustrated again that CNDs were most likely within the silk fibroin and were maintained a certain content during the process of concentration and spinning, causing the modified silk to become intrinsically blue fluorescent.

### Mechanical Properties of CNDs-Modified Silks

An important fact is that the CNDs-modified silks exhibited dramatically improved mechanical properties compared with control silk (Table 1 and Fig. S4). The breaking strength and elongation of 521.9 MPa and 19.2% for CNDs-1.25 silk significantly exceed those of control silk with 336.5 MPa and 12.5%, indicating that super-strong fluorescent silks could be fabricated using even small quantities of CNDs. Hence, 1.25 wt% was chosen as the maximum content of CNDs in this study to save the additional costs of further scale production and avoid any adverse effect of excessive CNDs on silkworms and silks [23]. Similar to other modified silks from silkworms fed silver nanoparticle [29], threonine [30], or nanohydroxyapatite powers [31], the mechanical properties of CNDs-modified silks were more variable than those of control silk. This may be attributed to the effect of exogenous addition on silkworm’s spinning behavior including spinning speed, which significantly determines the performance of silk [32]. Detailed investigations are still required to answer this question further. Moreover, the multi-functional silk with intrinsic fluorescence and enhanced mechanical properties was reported here for the first time, differentiating them from other fluorescent silk [9, 13]. Note that the mechanical properties of silks increased as CNDs content increased over the range 0.75–1.25 wt%, different from the results of silk modified by graphene [33], and provided the potential to modulate the properties for different applications. In addition, the mechanical properties of the silks demonstrated that they were stronger than other reported fluorescent silk [9, 13], although it was still lower than some reported silks [34, 35]. It should be noted that the environment in which the silkworms are raised, the process for degumming and approaches for testing all add to the variability of properties measured. Hence, in this study we only compared the properties and discussed the structures of silks fabricated within the same conditions.

**Table 1:** Mechanical properties of intrinsically fluorescent degummed silk fibers

Sample	Breaking strength (MPa)	Breaking elongation (%)	Modulus (GPa)	Breaking energy (kJ kg^−1^)
Control	336.5 ± 27.0	12.5 ± 2.2	7.3 ± 1.7	21.6 ± 5.0
CNDs-0.75	390.4 ± 91.5	15.3 ± 3.2	8.3 ± 2.7	30.4 ± 7.5
CNDs-1.00	479.4 ± 86.7	15.8 ± 3.1	8.4 ± 2.5	36.8 ± 7.3
**CNDs-1.25**	**521.9 ± 82.7**	**19.2 ± 4.3**	**8.9 ± 2.2**	**51.4 ± 14.3**
Transgenic silk [9]	419.97 ± 20.04	21.02 ± 1.49	13.61 ± 0.80	–
Silk from silkworm fed on rhodamine [13]	406 – 454	23.7 – 26.5	–	–

### Reinforcing Mechanism of CNDs-Modified Silks

**Fig. 3:**
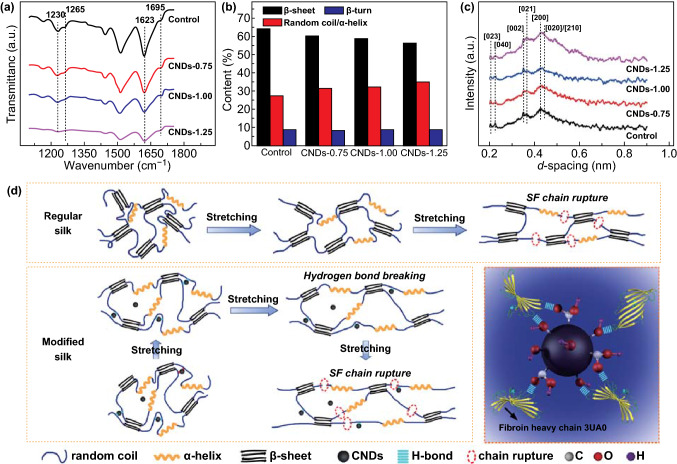
Crystalline structure and proposed reinforcing mechanism of multi-functional silk from silkworms fed CNDs. **a**–**c** FTIR spectra (**a**), content of secondary structures (**b**) obtained based on the amide I band in FTIR spectra, and 1D-WAXD patterns (**c**) of degummed silks. **d** Schematic diagram illustrating reinforcing mechanism of silks by incorporation of CNDs

We utilized FTIR to study the secondary structures of silk, strongly related to its mechanical properties. A peak at 1695 cm^−1^ was considered attributable to β-turn conformation [36], and the peaks at 1623 and 1230 cm^−1^ were related to β-sheet conformation [37]. The peak centered at 1265 cm^−1^ was assigned to a random coil/α-helix conformation [25]. The above characteristic peaks of silk fibroin were observed in all degummed silks, with no significant differences observed, suggesting that there were no strong covalent interactions between silk fibroin and CNDs. However, compared with control silk, the CNDs-modified silks contained a greater number of chains in random coil/α-helix conformation and fewer with β-sheet conformation (Figs. 3b and S5). This may be attributed to the abundant carboxyl and hydroxyl on the surface of CNDs (Fig. S6), favoring the formation of hydrogen bonds with amino groups of silk fibroin, hindering the transformation from random coil/α-helix to β-sheets.

The crystalline structure and orientation of silk fibers can be evaluated using synchrotron radiation wide-angle X-ray diffraction (SR-WAXD) (Figs. 3c and S7). All the silks exhibited the principal crystalline peaks of [002], [021], [200], and [020]/[210] lattice planes at d-spacing of 0.35, 0.37, 0.43, and 0.45 nm, with no remarkable differences, as shown in 1D WAXD. However, quantitative analyses of 1D WAXD revealed that the crystallinity of the modified silks was lower than control silks and decreased with increasing content of CNDs (Table 2), consistent with the FTIR result and previous reports [23, 38]. Based on “hydrogen bond barrier” effect [39] and nanoconfined crystallization as proposed by Pan et al. [27], the hydrogen bonds between CNDs and silk fibroin would have been expected to hinder the motion of silk fibroin, resulting in more amorphous. The β-sheet crystalline structure in silk belongs to the orthorhombic system [40], and the crystallite size along *a, b*, and *c* directions is determined from [200]/[210], [020], and [002] planes. Here, the lattice axes *a* and *c* are perpendicular, and along to silk fibroin chains, the lattice axis *b* is perpendicular to the β-sheet. The crystallite sizes of CNDs-modified silks in the *a* and *c* directions were slightly smaller than those of control silk. However, this was contrary to the result measured in the *b* direction. In addition, the crystalline volume evaluated by *L*_*a*_× *L*_*b*_× *L*_*c*_ [41] decreased with increasing the content of CNDs, significantly different from the modified silks from silkworms fed TiO_2_ [23]. This might be explained by differences in the size and dispersibility of CNDs and TiO_2_. At the same addition, compared with TiO_2_ with poor dispersibility and a larger size (20–50 nm), the smaller-sized CNDs (1–5 nm, Fig. S2) could homogeneously disperse over every segment of silk gland (Fig. 2c′–e′) and move along the protein molecules during the process of spinning. Hence, hydrogen bond interactions between silk fibroin and CNDs acted as a “cross-linked knot” and hindered the movement of molecular chains, confining crystallization of the silk fibroin. This tendency had already been observed in other polymers due to the efficient suppression of the crystal extension at higher concentration of additives [42, 43]. Furthermore, the smaller size and better mobility of CNDs favored the arrangement of molecular chains and phases that resulted in a comparable crystal orientation and higher mesophase orientation (Table 2), which included the oriented amorphous and interface zones between silk fibroin and CNDs [38, 44].

**Table 2:** Herman’s orientation function and crystallinity parameters of degummed silks

Sample	*f*_crystal_	*f*_mesophase_	Crystallinity (%)	Mesophase content (%)	Crystallite size (nm)		
					(200)/(210)	(020)	(002)
Control	0.9699	0.8579	49.3	13.1	5.0	3.1	10.4
CNDs-0.75	0.9680	0.8587	45.8	14.5	4.4	3.8	9.3
CNDs-1.00	0.9684	0.8613	44.5	14.9	4.1	3.9	9.7
CNDs-1.25	0.9677	0.8709	43.1	15.7	4.2	3.3	9.2

Based on the observation above, we hypothesize that the addition of CNDs would hinder the transformation of conformation, confine the crystallization, and induce orientation of mesophase. Those factors have played an important role in reinforcing the mechanical properties. Moreover, we should note that the addition of CNDs implied providing strong and stiff nanomaterial for the silk [45], further enhancing the modified silk. The proposed reinforcing mechanism is shown in Fig. 3d. Upon stretching, easily movable chains in random coil/α-helix conformation in the amorphous phase were first to deform. Meanwhile, the spherical morphology, nanometer size scale, and intensive hydrogen bond interactions caused the CNDs to move with protein chains [46], in turn providing more space for chains to move. This collaborative mobility endowed larger elongation to the modified silk fibers. As deformation increased, the relatively weak hydrogen bonds between CNDs and silk fibroin were broken first and dissipated energy [47]. In addition, the CNDs themselves provided stiffness and induced a transfer of stress from the silk to CNDs due to the nanofiller effect [45, 48]. Furthermore, the more content and higher orientation of the mesophase further reinforced the mechanical properties of CNDs-modified silk fibers.

### Non-cytotoxicity and Application of CNDs-Modified Silks

**Fig. 4:**
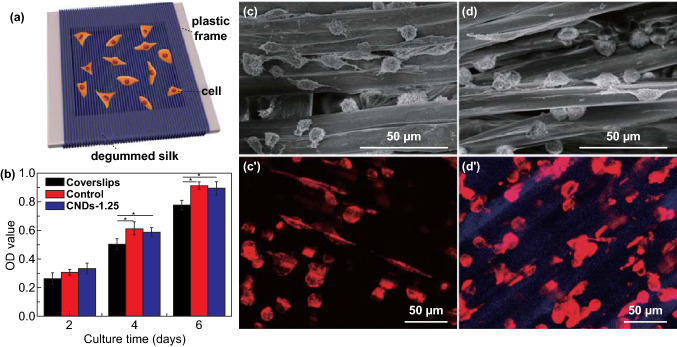
Growth and proliferation of Schwann cells (SCs) on multi-functional silks. **a** SCs cultured on silk scaffolds, constructed by wrapping silk around a hollow plastic frame. **b** OD value of SCs cultured on different substrates (coverslips, control, and CNDs-1.25 silk scaffolds) after 2, 4, 6 days. **c**–**d**′ SEM images (**c**, **d**) and LSCM images (**c**′, **d**′) of SCs after culturing for 4 days on control (**c, c**′) and CNDs-1.25 silk scaffolds (**d**, **d**′)

As a natural biomaterial, silk from normal silkworm has been widely used as tissue engineering scaffold. In this study, we constructed silk scaffolds by wrapping the fibers around a hollow plastic frame in order to culture SCs (Fig. 4a). After seeding for 2 days, the optical density (OD) value of SCs on different substrates was similar (Fig. 4b). After 4 or 6 days, SCs displayed higher proliferation on the silk scaffolds than coverslips, but no significant difference was observed between the control and modified scaffolds (Fig. 4c, d). This indicated that the non-cytotoxicity of natural silk was preserved in multi-functional silk scaffolds modified with CNDs and further confirmed by LSCM images of SCs stained with phalloidin (Fig. 4c′, d′). Furthermore, an important feature of fluorescent silk in tissue scaffolds is the improved visualization of cells. SCs which were in directly contact with fluorescent silks appeared bright pink, while other SCs appeared red. The blue fluorescence of silks also allowed more convenient monitoring of scaffold degradation. Taken together, CNDs-modified silks with excellent mechanical property, intrinsically fluorescent property, and non-cytotoxicity might motivate to open up new applications in tissue engineering field.

## Conclusions

In summary, multi-functional silks with reinforced mechanical properties, intrinsically fluorescence, and non-cytotoxicity can be produced simply using a simple in vivo modification method. The breaking strength and elongation of CNDs-1.25 fluorescent silk reached up to 521.9 ± 82.7 MPa and 19.2 ± 4.3%, respectively, considerably higher than that of regular silk and comparable to silks modified through post-treatment. This may be attributed to the hydrogen bonds between CNDs and silk fibroin, resulting in more random coil/α-helix structure, mesophase, and higher orientation. In addition, the nanosize and excellent dispersibility of CNDs were favored in the production of silks with a homogeneous blue fluorescence. It can be expected that large-scale production of multi-functional silks would be feasible and that the use of such silk as active scaffolds in tissue engineering would improve the functionalities of conventional biomaterials.

## Electronic supplementary material

Below is the link to the electronic supplementary material.

Supplementary material 1 (PDF 822 kb)
