# Supplementary material for: Super-strong and Intrinsically Fluorescent Silkworm Silk from Carbon Nanodots Feeding
Source: Nanomicro Lett. 2019 Sep 11;11:75. doi: 10.1007/s40820-019-0303-z (PMC7770652; doi:10.1007/s40820-019-0303-z)
Supplement: Supplementary file 1 — Supplementary material 1 (PDF 822 kb) [file 40820_2019_303_MOESM1_ESM.pdf]

Supporting Information for

Super-strong and Intrinsically Fluorescent Silkworm Silk from Carbon Nanodots Feeding

Suna Fan[1], Xiaoting Zheng[1], Qi Zhan[1], Huihui Zhang[1], Huili Shao[1], Jiexin Wang[2], Chengbo Cao[3, 4,]*, Meifang Zhu[1], Dan Wang[2,] *, Yaopeng Zhang[1,] *

[1]State Key Laboratory for Modification of Chemical Fibers and Polymer Materials, Shanghai Belt and Road Joint Laboratory of Advanced Fiber and Low-Dimension Materials, College of Materials Science and Engineering, Donghua University, Shanghai 201620, People's Republic of China

[2]State Key Laboratory of Organic-Inorganic Composites, Beijing University of Chemical Technology, Beijing 100029, People's Republic of China

[3]School of Chemistry and Chemical Engineering, Shandong University, Jinan 250100, People's Republic of China

[4]School of Chemistry and Chemical Engineering, Yantai University, Yantai 264005, People's Republic of China

*Corresponding authors. E-mail: zyp@dhu.edu.cn (Yaopeng Zhang); wangdan@mail.buct.edu.cn (Dan Wang); cbcao@sdu.edu.cn (Chengbo Cao)

Supplementary Figures

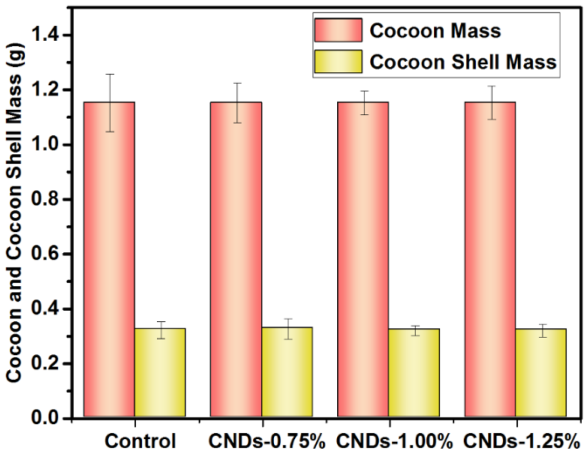

Fig. S1 The mass of single cocoon and cocoon shell. All the cocoons have the similar mass, indicating that the addition of CNDs in the diets is safe for silkworm.

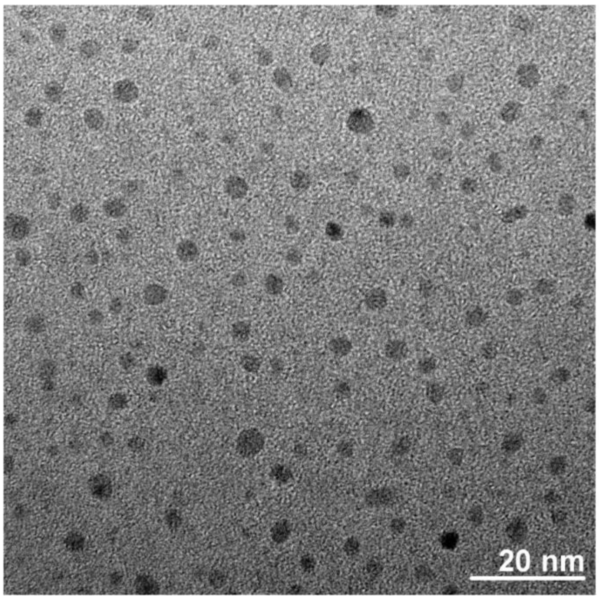

Fig. S2 TEM image of CNDs. The CNDs have diameters ranging from 1 to 5 nm, and separated from each other, illustrating good dispersibility in aqueous solution

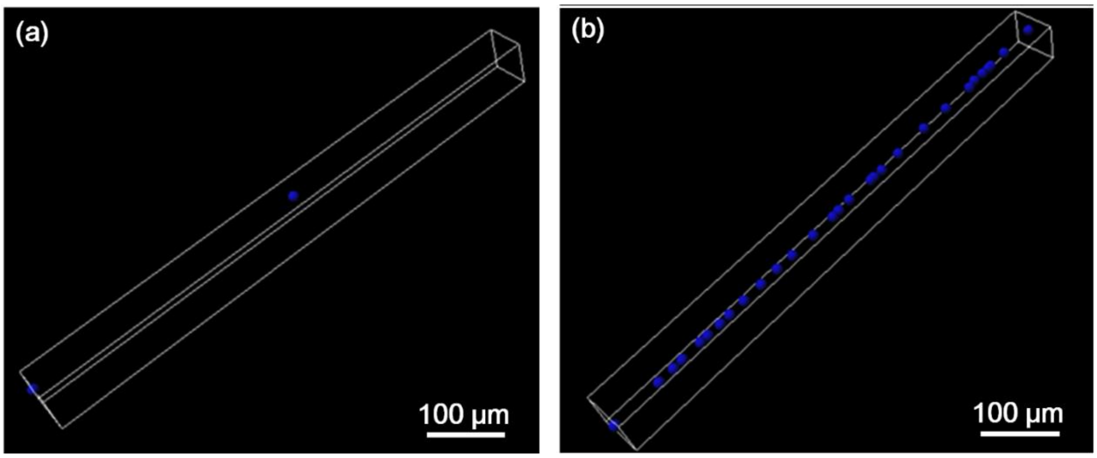

Fig. S3 3D reconstructed CLSM images of **a** control and **b** modified degummed silk. These results were consistent with 2D CLSM images (Fig. 2) and suggested the fluorescence uniformity of modified silk.

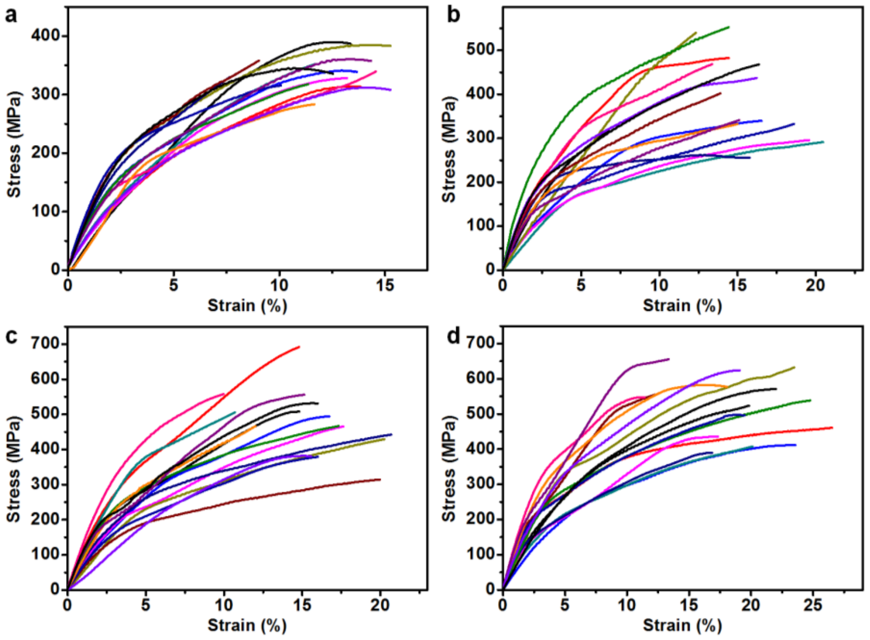

Fig. S4 Stress-strain curves of different silk fibers: **a** Control, **b** CNDs-0.75%, **c** CNDs-1.00%, **d** CNDs-1.25%. Compared to control silks, the breaking strength and elongation of CNDs modified silks increased significantly. In addition, the mechanical properties reinforced gradually with increasing the addition of CNDs.

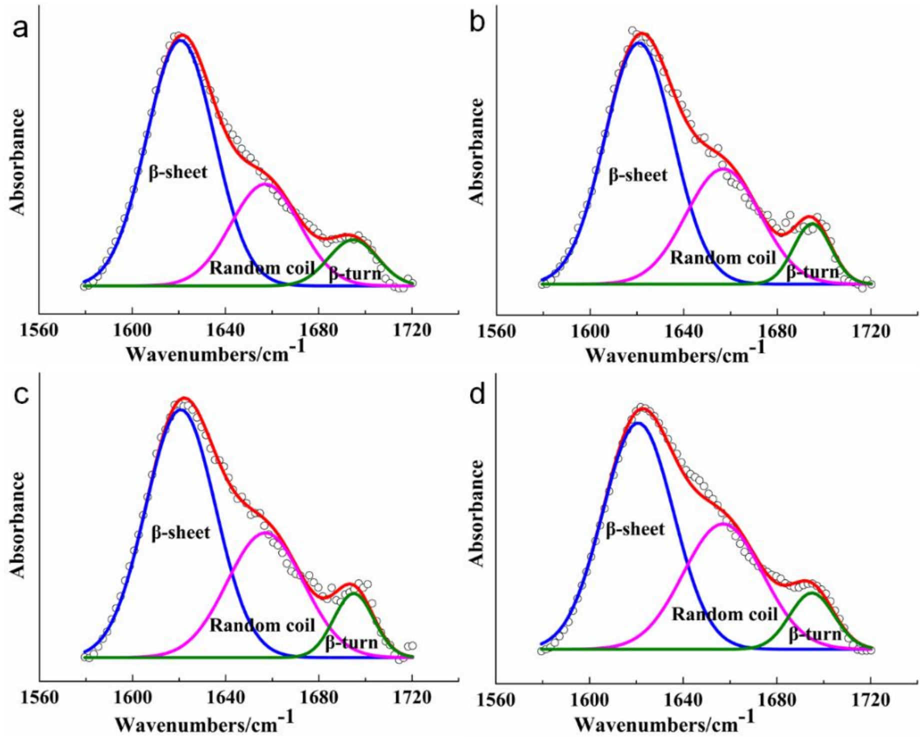

Fig. S5 The deconvolution of FTIR spectra in amide I band of different silk fibers. **a** Control, **b** CNDs-0.75%, **c** CNDs-1.00%, **d** CNDs-1.25%

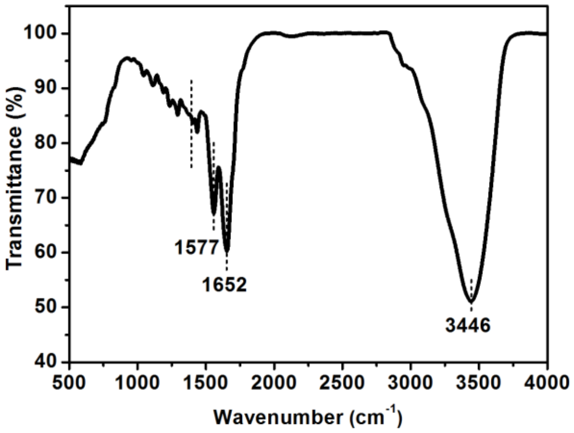

Fig. S6 FTIR spectra of CNDs. The absorption peaks at 1577, 1652, and 3446 cm^{-1} were ascribed to the bending vibrations of N-H, asymmetric stretching vibration of C=O, and stretching vibration of C-OH [S1, S2]. This indicated that there were abundant carboxyl and hydroxyl on the surface of CNDs.

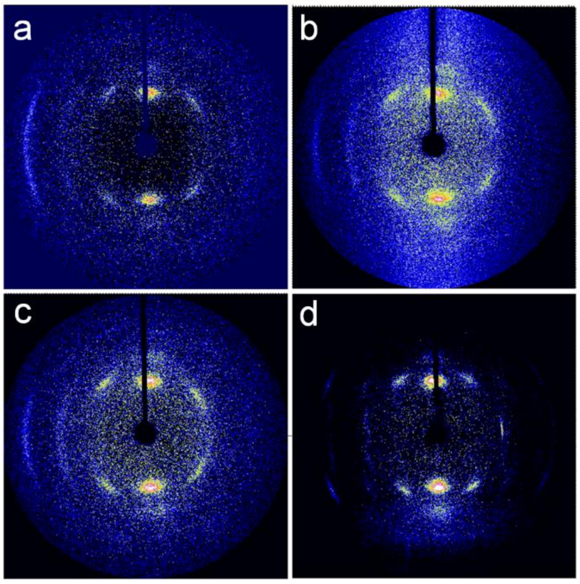

Fig. S7 Two-dimensional SR-WAXD patterns of different silk fibers. **a** Control, **b** CNDs-0.75%, **c** CNDs-1.00%, **d** CNDs-1.25%

Supplementary References

[S1] S. Zhu, Q. Meng, L. Wang, J. Zhang, Y. Song et al., Highly photoluminescent carbon dots for multicolor patterning, sensors, and bioimaging. Angew. Chem. Int. Ed. **52**, 3953-3957 (2013). https://doi.org/10.1002/anie.201300519

[S2] S. Lu, L. Sui, J. Liu, S. Zhu, A. Chen, M. Jin, B. Yang, Near-infrared photoluminescent polymer-carbon nanodots with two-photon fluorescence. Adv. Mater. **29**, 1603443 (2017). https://doi.org/10.1002/adma.201603443
